# A Common KIF6 Polymorphism Increases Vulnerability to Low-Density Lipoprotein Cholesterol: Two Meta-Analyses and a Meta-Regression Analysis

**DOI:** 10.1371/journal.pone.0028834

**Published:** 2011-12-21

**Authors:** Brian A. Ference, Wonsuk Yoo, John M. Flack, Michael Clarke

**Affiliations:** 1 Division of Translational Research and Clinical Epidemiology, Department of Internal Medicine, Wayne State University School of Medicine, Detroit, Michigan, United States of America; 2 Centre for Evidence Based Medicine, University of Oxford, Oxford, United Kingdom; 3 Division of Biostatistics and Epidemiology, University of Tennessee Health Sciences Center, Memphis, Tennessee, United States of America; 4 Clinical Trial Service Unit, University of Oxford, Oxford, United Kingdom; 5 All-Ireland Hub for Trials Methodology Research, Queen's University Belfast, Belfast, United Kingdom; John Hopkins Bloomerg School of Public Health, United States of America

## Abstract

**Background:**

We sought to determine if a common polymorphism can influence vulnerability to LDL cholesterol, and thereby influence the clinical benefit derived from therapies that reduce LDL cholesterol.

**Methods:**

We conducted a meta-analysis of the association between a common Trp719Arg polymorphism in the kinesin-like protein 6 (KIF6) gene and the risk of cardiovascular disease (CVD), and a meta-regression analysis to measure the effect modification of this polymorphism on the association between LDL cholesterol and the risk of CVD. We used this measure of genetic effect modification to predict the expected difference in clinical benefit among KIF6 719Arg allele carriers and non-carriers in response to therapies that reduce LDL cholesterol. We then conducted a meta-analysis of statin trials to compare the expected difference in clinical benefit with the observed difference during treatment with a statin.

**Results:**

In a meta-analysis involving 144,931 participants, the KIF6 719Arg allele was not associated with the relative risk (RR) of CVD (RR: 1.02, 95%CI: 0.98–1.07, p = 0.288). Meta-regression analysis involving 88,535 participants, however, showed that the 719Arg allele appears to influence the effect of LDL cholesterol on the risk of CVD. KIF6 carriers experienced a 13% greater reduction in the risk of CVD per mmol/L decrease in LDL cholesterol than non-carriers. We interpreted this difference as the expected difference in clinical benefit among KIF6 carriers and non-carriers in response to therapies that lower LDL cholesterol. The difference in clinical benefit predicted by the increased vulnerability to LDL cholesterol among KIF6 carriers (ratio of RR: 0.87, 95%CI: 0.80–0.94, p = 0.001) agreed very closely with the observed difference among 50,060 KIF6 carriers and non-carriers enrolled in 8 randomized trials of statin therapy (ratio of RR: 0.87, 95%CI: 0.77–0.99, p = 0.038).

**Conclusion:**

The KIF6 719Arg allele increases vulnerability to LDL cholesterol and thereby influences the expected clinical benefit of therapies that reduce LDL cholesterol.

## Introduction

A common single nucleotide polymorphism (rs20455) results in an argine to tryptophan substitution at position 719 (Trp719Arg) in the gene encoding kinesin-like protein 6 (KIF6). Some [1]–[4], but not all [5], [6], genetic sub-studies of previously conducted randomized trials of statin therapy have reported that persons who inherit one or more copies of the 719Arg allele experienced a greater reduction in clinical events than non-carriers during treatment with a statin, despite the fact that both carriers and non-carriers experienced similar reductions in low-density lipoprotein (LDL) cholesterol during treatment [4]–[6]. These findings have generated considerable controversy because it is not clear how carriers of the 719Arg allele could experience a greater clinical benefit than non-carriers in response to the same magnitude of LDL cholesterol reduction [7].

One possible explanation is that the KIF6 719Arg allele may increase the risk of cardiovascular disease (CVD), and that treatment with a statin may ameliorate this increased risk through a pleiotropic effect [8], [9]. Indeed, this hypothesis is consistent with the observation that the KIF6 719Arg allele was associated with an increased risk of CVD in three large prospective epidemiologic cohort studies [10]–[13]. A subsequent meta-analysis of 19 case-control studies, however, failed to find any association between this polymorphism and the risk of CVD [14]. The question thus arises as to whether it is biologically plausible that carriers of a 719Arg allele could experience a greater clinical benefit from treatment with a statin than non-carriers, if this polymorphism does not have any impact on the LDL cholesterol lowering effect of statins, and if this polymorphism is not associated with an increased risk of CVD.

A biologically plausible hypothesis that can potentially reconcile these apparently conflicting findings is that the KIF6 719Arg allele may increase vulnerability to LDL cholesterol. If carriers of the 719Arg allele are more vulnerable to the deleterious effects of LDL cholesterol, then they should experience a greater increase in the risk of CVD per unit increase in LDL, and a correspondingly greater reduction in the risk of CVD per unit decrease in LDL, as compared to non-carriers. Therefore, if KIF6 carriers are more biologically vulnerable to LDL cholesterol, they should derive a greater clinical benefit per unit reduction in LDL cholesterol than non-carriers in response to therapies that lower serum LDL cholesterol levels.

The primary aim of our study was to test the hypothesis that a common polymorphism can influence biological vulnerability to LDL cholesterol, and thereby influence the expected clinical benefit of therapies that reduce serum LDL cholesterol levels. To test this hypothesis, we first conducted a systematic review and meta-analysis of the association between the KIF6 719Arg allele and the risk of CVD. We then conducted a meta-regression analysis to measure the effect of the KIF6 719Arg allele on the association between LDL cholesterol on the risk of CVD. We used this measure of effect modification to estimate the expected difference in clinical benefit among KIF6 carriers and non-carriers in response to therapies that lower serum LDL cholesterol level. We then conducted a systematic review and meta-analysis of the effect of the KIF6 719Arg allele on the clinical efficacy of statin therapy. Finally, we compared the expected difference in clinical benefit predicted by the meta-regression analysis with the observed difference in clinical benefit among KIF6 carriers and non-carriers in the meta-analysis of statin trials.

## Methods

We searched Pubmed, Embase, Index of Science, HUGEnet, and Google Scholar, using the search terms: KIF6, rs20455, 719Arg, and Trp719Arg without language restriction; we also checked the abstracts and presentations from major cardiovascular medicine meetings held from 2004 to present [15], and searched the references of selected articles [16], in an attempt to identify all published and unpublished studies that evaluated either the association between the KIF6 Trp719Arg polymorphism and the risk of CVD, or the effect of this polymorphism on the clinical benefit of statin therapy. We defined KIF6 719Arg “carriers” as having the Arg/Arg or Arg/Trp genotypes, and “non-carriers” as having the Trp/Trp genotype. Because we did not have access to individual patient-level data for any of the studies included in this analysis, we could not harmonize the primary CVD outcome definition across studies. Therefore, the primary outcome for this analysis was CVD: defined as the study-specific primary CVD outcome definition in each of the included studies (which in some studies was a composite outcome). To minimize the potential for population stratification bias, we only included data on Caucasian subjects [17]. All data were extracted in duplicate and discrepancies were resolved by discussion.

First, we conducted an updated systematic review and meta-analysis of the association between KIF6 719Arg carrier status and the risk of CVD. For this analysis, we included case-control studies, epidemiologic cohort studies, and the treatment allocation arms of randomized trials of statin therapy that have reported data separately among KIF6 719Arg allele carriers and non-carriers, but excluded family-based studies. We included both the placebo allocation and statin allocation arms of the placebo controlled trials, as well as both the less intensive and more intensive treatment allocation arms of the trials that compared different treatment intensities of statin therapy. Each treatment allocation arm was considered as a separate prospective cohort study for the purposes of this analysis. For study samples that were included in more than one report, we used data from only the most recent report. In each study, Hardy-Weinberg equilibrium was assessed, as appropriate, using a chi-square test. For each prospective cohort study (including the treatment allocation arms of the randomized trials), we extracted the total number of KIF6 719Arg allele carriers and non-carriers, as well as the number of KIF6 carriers and non-carriers who experienced an incident CVD event during study follow-up. From each case-control study, we extracted the total number of prevalent cases and controls for both KIF6 719Arg allele carriers and non-carriers. We used these data to create 2×2 tables to calculate the unadjusted relative risk (RR), and 95% confidence interval (CI), for each prospective cohort study, and the unadjusted odds ratio (OR), and 95% CI, for each case-control study (as an approximation of the RR) [18]. We combined these results to obtain a summary point estimate of the association between KIF6 719Arg allele carrier status and the risk of CVD using an inverse variance weighted random effects meta-analysis model [19]. We then repeated these analyses by KIF6 genotype.

Next, we performed a random effects meta-regression analysis to measure the effect modification of study baseline LDL cholesterol level on the association between KIF6 carrier status (and separately KIF6 genotype) and the risk of CVD [20]. For this analysis, we included all prospective epidemiologic cohort studies that provided information on baseline average serum LDL cholesterol level of the population under study, and the treatment allocation arms of the randomized trials (each considered as a separate cohort). For each treatment allocation arm of the statin trials, we defined baseline LDL cholesterol as the average LDL cholesterol level during study follow-up. If this information was not available, we used the LDL cholesterol level measured at one year follow-up [21], [22]. We considered only incident CVD events in each study for this analysis. The amount of between-study variance explained (R^2^) by the effect modification was calculated as the difference in tau^2^ between the regression model with and without a regression term, expressed as a percentage.

Finally, we conducted an updated systematic review and meta-analysis of statin trials that have reported results stratified by KIF6 carrier status (or KIF6 genotype). From the report of each randomized trial, we extracted data on the number of persons assigned to either treatment allocation arm and the number of incident CVD events that occurred during study follow-up in each arm, stratified by KIF6 carrier status. We used these data to create 2×2 tables to calculate an unadjusted RR (and 95% CI) for the effect of statin therapy as compared to placebo (or more intensive statin therapy as compared to less intensive statin therapy) on the risk of incident CVD events, separately among KIF6 carriers and non-carriers in each study. All analyses were by the intent-to-treat principle. We then standardized the estimate of the effect of statin therapy per mmol/L reduction in LDL cholesterol across trials by multiplying the natural logarithm of the RR for each trial (and its standard error) by 1÷ Y, where Y is difference in average LDL cholesterol during follow-up between the two treatment allocation arms (or, where this information wasn't available, the difference in average LDL cholesterol at one year follow-up). We then combined the adjusted RR for each trial using an inverse variance weighted random effects model [19], separately among KIF6 carriers and non-carriers, Finally, we compared the summary estimates of the effect of statin therapy per mmol/L reduction in LDL cholesterol among KIF6 carriers and non-carriers using both a Z-test [23], and random effects meta-regression analysis [20].

We standardized the effect of statin therapy per mmol/L reduction in LDL cholesterol in order to compare the observed difference in clinical benefit in the meta-analysis with the expected difference in clinical benefit estimated from the meta-regression analysis using the same scale. Standardizing the effect of statin therapy also allowed us to include both the trials that compared statin with placebo and the trials that compared more intensive and less intensive statin therapy. In the meta-analysis, therefore, the primary measure of effect is not the clinical benefit of statin therapy per se, but rather the clinical benefit per mmol/L reduction in LDL cholesterol during treatment with a statin.

Heterogeneity was measured using Cochran's Q, and the I^2^ metric [24]. All statistical tests used a two-sided α<0.05 as the threshold for statistical significance, and all analyses were performed using Stata (version 10.1).

## Results

**Figure 1:**
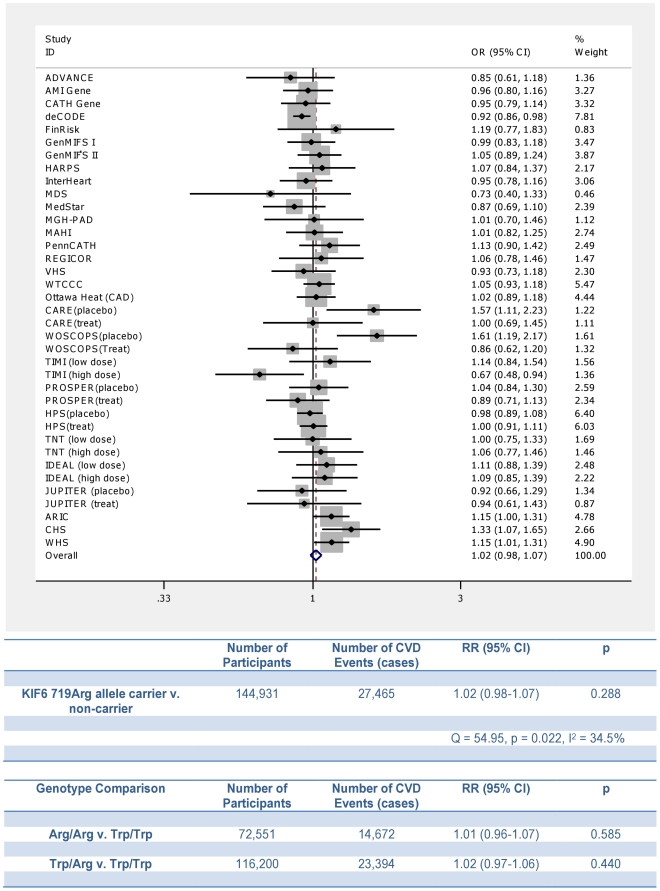
Meta-Analysis of the association between KIF6 719Arg allele and the risk of cardiovascular disease. Squares represent the point estimate of effect for each study, and horizontal lines through the squares represent 95% CIs. The area of each square reflects the weight assigned to that study. The center of the diamond represents the summary point estimate of effect, and the width of the diamond represents the 95% CI of the summary point estimate of effect. Each study included in the analysis is described in Table S1.

A total of 37 case-control studies, prospective cohort studies, or randomized trial treatment allocation arms (each considered as a separate cohort), including 144,931 participants and 27,465 CVD events, were included in our meta-analysis of the association between the KIF6 719Arg allele and the risk of CVD (Table S1) [1]–[6], [10]–[13], [14], [25]–[33]. Figure 1 shows that among the included studies, KIF6 719Arg allele carrier status was not associated with an increased risk of CVD (RR: 1.02, 95% CI: 0.98–1.07, p = 0.288). Similarly, we found no association between KIF6 genotype and the risk of CVD (719Arg allele homozygote RR: 1.01, 95% CI: 0.96–1.07, p = 0.585; and 719Arg allele heterozygote RR: 1.02, 95% CI: 0.97–1.106, p = 0.440). These results did not change appreciably in multiple sensitivity analyses, including analyses that excluded all active treatment allocation arms of the statin trials (719Arg carrier RR: 1.04, 95% CI: 0.98–1.09, p = 0.176).

**Figure 2:**
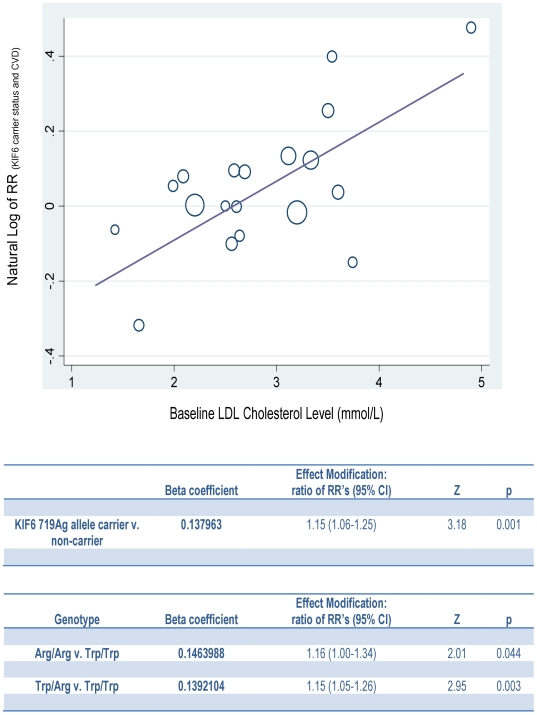
Meta-Regression of effect of baseline LDL cholesterol on the association between KIF6 and risk of cardiovascular disease. For each study, the natural logarithm of RR for the association between KIF6 carrier status and the risk of CVD is plotted against the baseline mean LDL cholesterol level of the population under study. Area of circles indicates weight assigned to each study according to the inverse of the study-specific standard error. Line represents the fitted regression line that minimizes the weighted sum of the squared errors.

Among the 37 study samples included in our meta-analysis, 19 prospective cohort studies (including all 16 treatment allocation arms of the randomized trials, each considered as a separate cohort) involving a total of 88,535 participants and 9,692 incident CVD events, provided information on the average baseline LDL cholesterol level of the population under study and therefore were included in our meta-regression analysis [1]–[6], [10]–[13], [14], [25]. Figure 2 shows the natural logarithm of the RR for the association between KIF6 719Arg allele carrier status and the risk of CVD plotted against the baseline LDL cholesterol level for each study included in this analysis. Meta-regression demonstrated that the association between KIF6 carrier status and the risk of CVD varied significantly by study baseline LDL cholesterol level. The RR for the association between KIF6 719Arg carrier status and the risk of CVD increased by 1.15-fold for each mmol/L higher baseline LDL cholesterol level of the population under study (ratio of RR: 1.15, 95% CI: 1.06–1.25, p = 0.001). The effect modification of LDL cholesterol on the association between the KIF6 719Arg allele and the risk of CVD was similar among homozygous 719Arg allele carriers (ratio of RR: 1.16, 95% CI: 1.00–1.34, p = 0.044) and heterozygous carriers (ratio of RR: 1.15, 95% CI: 1.05–1.26, p = 0.003). This effect modification explained approximately 60% of the excess between-study heterogeneity (R^2^ = 58.7%).

Relying on the symmetrical nature of the effect modification between any two epidemiologic exposures [34], we used the meta-regression analysis results to also measure the effect modification of KIF6 carrier status on the association between LDL cholesterol and the risk of CVD (Text S1). We found that for each mmol/L increase in LDL cholesterol, KIF6 719Arg carriers experienced a 15% greater increase in the relative risk of CVD as compared to non-carriers (ratio of RR: 1.15, 95% CI: 1.06–1.25, p = 0.001). Similarly, for each mmol/L decrease in LDL cholesterol, KIF6 719Arg carriers experienced a corresponding 13% greater reduction in the relative risk of CVD (ratio of RR: 0.87, 95% CI: 0.80–0.94, p = 0.001). The evidence for effect modification of KIF6 carrier status on the association between LDL cholesterol and the risk of CVD was robust, and remained very consistent in multiple sensitivity analyses, including analyses that excluded data from the statin trials (Table 1). We interpreted the ratio of the relative risks for a one mmol/L decrease in LDL cholesterol as the expected difference in clinical benefit among KIF6 carriers and non-carriers in response to therapies that lower LDL cholesterol. The results of the meta-regression analysis thus predict that KIF6 carriers should experience an approximately 13% greater relative clinical benefit per mmol/L reduction in LDL than non-carriers in response to therapies that lower serum LDL cholesterol level.

**Table 1:** Sensitivity analysis of the effect modification of KIF6 on the association between LDL and risk of CVD.

Studies included in the Analysis	Number of Studies	Number of Participants	Number of CVD Events	Effect Modification:Ratio of RR's (95% CI)	p
All	19	88,535	9,692	0.87 (0.80–0.94)	0.001
*Prospective cohort studies (excluding the statin trials)	3	38,475	2,385	0.81 (0.46–1.41)	0.455
Placebo allocation arms of the statin trials	5	18,568	3,298	0.76 (0.64–0.89)	0.001
Prospective cohort studies and placebo allocation arms of the statin trials	8	57,043	5,683	0.78 (0.65–0.94)	0.009
Control arms of the statin trials (placebo and less Intense statin allocation arms)	8	25,091	4,087	0.84 (0.72–0.98)	0.029
Prospective cohort studies and control arms of the statin trials (placebo and less intense statin allocation arms)	11	63,566	6,472	0.84 (0.72–0.97)	0.015
Prospective cohort studies and active treatment arms of the statin trials (statin and more intense statin allocation arms)	11	63,444	5,602	0.88 (0.81–0.96)	0.003

*Prospective cohort studies include: Atherosclerosis Risk in Communities study, Cardiovascular Health Study, and Women's Heath Study.

**Figure 3:**
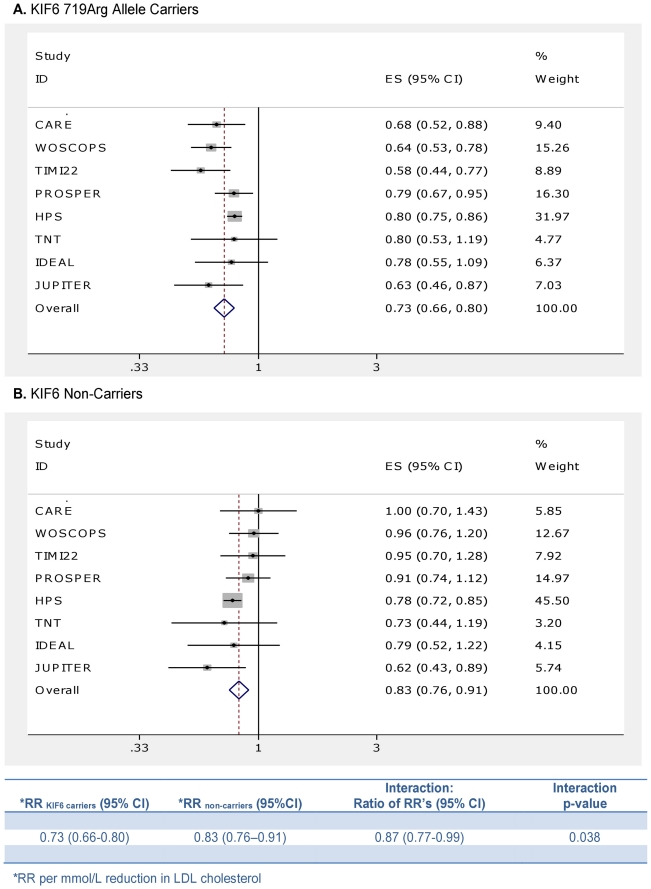
Meta-Analysis of the clinical benefit of statin therapy stratified by KIF6 carrier status. Symbols and conventions as described in Figure 1. The baseline characteristics of each study included in this analysis is provided in Table S2.

A total of 8 randomized trials, involving 50,060 participants and 7,307 incident cardiovascular events, were included in our meta-analysis of the effect of statin therapy, stratified by KIF6 carrier status (Table S2) [1]–[6], [25]. Figure 3 shows that among 719Arg allele carriers, treatment with a statin as compared to placebo (or more intensive statin therapy as compared to less intensive statin therapy) resulted in a 27% reduction in the risk of CVD events per mmol/L reduction in LDL cholesterol (RR: 0.73, 95% CI: 0.66–0.80, p<0.0001); and among non-carriers, this treatment resulted in a 17% reduction in the risk of CVD events per mmol/L reduction in LDL cholesterol (RR: 0.83, 95% CI: 0.76–0.91, p<0.0001). The ratio of these two risk ratios exceeded the pre-defined α threshold for this study (ratio of RR: 0.87, 95% CI: 0.77–0.99, p = 0.038), thus indicating that KIF6 719Arg carriers derived a greater clinical benefit for each mmol/L reduction in LDL cholesterol during treatment with a statin than did non-carriers. The results were nearly identical when we estimated the effect modification of KIF6 carrier status on the clinical benefit of statin therapy per mmol/L reduction in LDL cholesterol using meta-regression (ratio of RR: 0.87, 95% CI: 0.76–0.99, p = 0.040).

The magnitude of the difference in clinical benefit per mmol/L reduction in LDL cholesterol observed among KIF6 719Arg carriers and non-carriers in response to treatment with a statin (ratio of RR: 0.87, 95% CI: 0.77–0.99, p = 0.038) was nearly identical with the expected difference in clinical benefit per mmol/L reduction in LDL cholesterol predicted by the increased vulnerability to LDL cholesterol among KIF6 719Arg carriers from the meta-regression analysis (ratio of RR: 0.87, 95% CI: 0.80–0.94, p = 0.001).

## Discussion

Our study demonstrates that the common KIF6 719Arg allele appears to increase vulnerability to LDL cholesterol, and thereby influences the expected clinical benefit from therapies that lower serum LDL cholesterol levels. Persons who inherit one or more copies of the 719Arg allele appear to be more biologically vulnerable to the deleterious effects of LDL cholesterol than non-carriers. As a result, KIF6 719Arg carriers appear to derive a greater reduction in the risk of CVD per mmol/L reduction in LDL cholesterol than non-carriers. The results of our study resolve several apparently conflicting lines of evidence to provide a biologically plausible explanation for how KIF6 carriers can derive a greater clinical benefit from treatment with a statin than non-carriers, even though this polymorphism does not have any impact on the LDL cholesterol lowering effect of statins, and is not independently associated with the risk of CVD.

**Figure 4:**
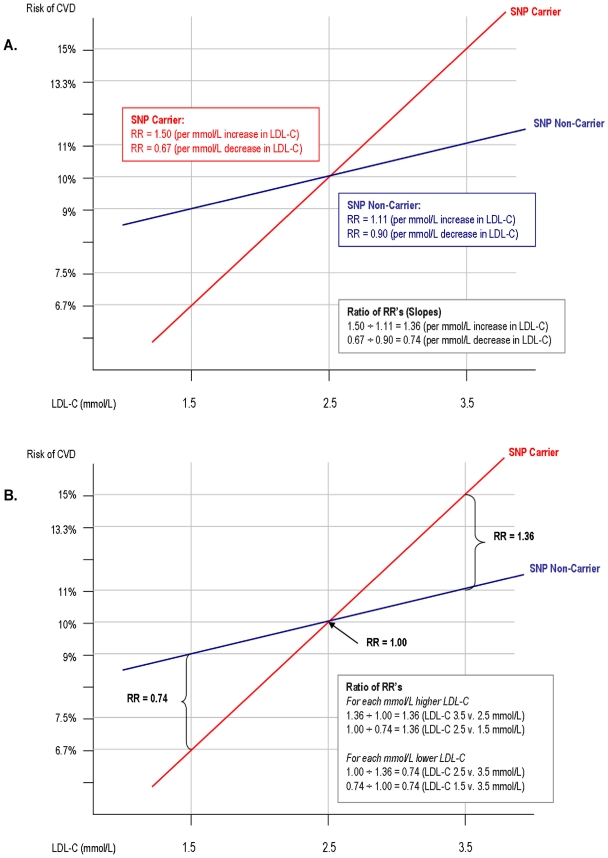
Epidemiological characteristics of a polymorphism that increases vulnerability to LDL cholesterol. *Panel A: Effect of SNP carrier status on the association between LDL cholesterol and risk of CVD.* Panel A shows the effect of a one mmol/L change in LDL cholesterol (LDL-C) on the risk of CVD among SNP carriers and non-carriers. The slope of each line represents the relative risk (RR) of CVD associated with a one mmol/L change in LDL-C. The slope (RR) is greater for carriers of a SNP that increases vulnerability to LDL-C indicating a greater increase in the RR of CVD per unit increase in LDL-C, and a corresponding greater reduction in the RR per unit decrease in LDL-C among SNP carriers as compared to non-carriers. The ratio of the slopes (ratio of RR's) is constant and is a measure of effect modification of SNP carrier status on the association between LDL-C and the risk of CVD. *Panel B: Effect of LDL cholesterol on the association between SNP carrier status and risk of CVD.* Panel B shows that for a SNP that increases vulnerability to LDL-C, the association between that SNP and the risk of CVD will vary by LDL-C level. At higher LDL-C levels, the SNP will be positively associated with the risk of CVD, at moderate LDL-C levels it will have a null association with CVD, and at lower LDL-C levels the SNP will be inversely associated with the risk of CVD. The overall association between the SNP and the risk of CVD will therefore vary according to the average LDL-C level of the population(s) under study. The ratio of the RR for the association between SNP carrier status and the risk of CVD at any two LDL-C levels is a measure of the effect modification of LDL-C on the association between SNP carrier status and the risk of CVD. **NB:** It can be seen from the Figure that the ratio of the slopes (RR's) in Panel A is exactly equal to the ratio of RR's in Panel B. This observation demonstrates graphically that the magnitude the effect modification of SNP carrier status on the association between LDL-C and the risk of CVD (Panel A) is exactly equal to the magnitude of the effect modification of LDL-C on the association between SNP carrier status and the risk of CVD (Panel B).

More generally, our study demonstrates that a common polymorphism can influence vulnerability to LDL cholesterol, and thereby influence the expected clinical benefit from therapies designed to lower serum levels of LDL cholesterol. The concept of genetically-mediated differential vulnerability to LDL cholesterol is illustrated in Figure 4. The Figure demonstrates graphically that persons who inherit a polymorphism that increases vulnerability to LDL cholesterol experience a greater increase in the risk of CVD per unit increase in LDL cholesterol, and a correspondingly greater reduction in the risk of CVD per unit decrease in LDL cholesterol as compared to non-carriers. This difference in the reduction of CVD risk per unit decrease in LDL cholesterol can be interpreted as the difference in expected clinical benefit in response to therapies that lower serum LDL cholesterol level. The Figure also shows that if a polymorphism increases vulnerability to LDL cholesterol, then the association between that polymorphism and the risk of CVD will vary according to the average LDL cholesterol level of the population(s) under study, and can be positive, inverse, or null. The Figure thus illustrates graphically that persons who inherit a polymorphism that increases vulnerability to LDL cholesterol should experience a greater clinical benefit per unit reduction in LDL cholesterol than non-carriers in response to therapies that lower serum LDL levels, and this difference in clinical benefit will be independent of the association between that polymorphism and the risk of CVD.

Our study shows that, on average, KIF6 719Arg allele carrier status is not associated with the risk of CVD. Our study also shows, however, that this association varies significantly according to the average baseline LDL cholesterol level of the population under study (Figure 2). Furthermore, we found that persons who inherit one or more copies of the KIF6 719Arg allele experienced a 15% greater increase in the risk of CVD per mmol/L increase in LDL cholesterol, and a 13% greater reduction in the risk of CVD per mmol/L decrease in LDL cholesterol as compared to non-carriers. We interpreted this gene-by-environment interaction as evidence that the KIF6 719Arg allele increases a person's vulnerability to LDL cholesterol. Interpreting genetic interactions in this way has practical clinical implications. It implies that because KIF6 carriers are more vulnerable to the deleterious effects of LDL cholesterol, they should experience a greater clinical benefit per mmol/L reduction in LDL cholesterol than non-carriers in response to therapies that reduce serum levels of LDL cholesterol. We directly tested this interpretation of genetic effect modification by comparing the magnitude of the expected difference in clinical benefit predicted by the difference in vulnerability to LDL cholesterol with the observed difference in clinical benefit among KIF6 carriers and non-carriers during treatment with a statin. Consistent with the hypothesis that the KIF6 719Arg allele increase vulnerability to LDL cholesterol, we found that KIF6 carriers did indeed experience a slightly greater clinical benefit per mmol/L reduction in LDL cholesterol than did non-carriers. Furthermore, we found that the magnitude of the difference in clinical benefit during treatment with a statin was nearly identical to the magnitude of the difference in clinical benefit predicted by the difference in vulnerability to LDL cholesterol among KIF6 carriers and non-carriers. This close agreement between the observed and expected difference in clinical benefit substantially increases the validity of our finding that the KIF6 719Arg allele increases vulnerability to LDL cholesterol.

Our findings do not challenge the results of a large-scale meta-analysis of statin randomized trials that found that the clinical benefit of statin therapy is determined largely by the magnitude of LDL cholesterol reduction achieved during treatment [21], [22]. In our study, the clinical benefit of statin therapy among both KIF6 carriers and non-carriers appeared to be directly and linearly related to the magnitude of LDL reduction achieved during treatment. Because KIF6 719Arg allele carriers appear to be more vulnerable to the deleterious effects of LDL cholesterol, however, they appear to derive a greater clinical benefit per mmol/L reduction in LDL cholesterol during treatment with a statin than do non-carriers.

Importantly, our study shows that the KIF6 719Arg allele increases vulnerability to LDL cholesterol even though it is not, on average, associated with an increased risk of CVD. This means that all 2.5 million directly genotyped and imputed polymorphism measured in previously conducted genome-wide association studies of CVD, very few of which have been shown to be reliably associated with the risk of CVD [31], [35], can now be re-evaluated to determine if they influence vulnerability to LDL cholesterol. Using the approach outlined here, it may be possible to identify polymorphism that have a large impact on vulnerability to LDL cholesterol, and therefore have a correspondingly large influence on the expected clinical benefit from therapies used to lower serum LDL cholesterol levels. Our approach can be easily extended to search for polymorphism that influence vulnerability to other modifiable risk factors for CVD, and therefore influence the expected clinical benefit from therapies used to treat these risk factors. Using this approach, it may be possible to identify the risk factor(s) for CVD to which each person is most vulnerable based on their genomic background. Identifying and treating the risk factor(s) to which each person is most vulnerable may be a practical strategy to personalize the prevention of cardiovascular disease.

Our study has several limitations. Most importantly, we did not have any data on the association between LDL cholesterol and the risk of CVD, stratified by KIF6 carrier status. Therefore, we could not estimate the effect modification of KIF6 on the association between LDL cholesterol and the risk of CVD directly. Instead, we estimated this effect from the meta-regression estimate of the effect modification of LDL cholesterol on the association between the KIF6 719Arg allele and the risk of CVD. We believe this approach is valid because these two estimates of effect modification are equivalent as shown in Text S1. This can be demonstrated by observing that a meta-regression term and an interaction term in a multivariable model are merely different methods of estimating the same effect modification between any two exposures. Furthermore, a single interaction term in a multivariable model defines the effect modification between any two exposure variables. Therefore, the effect modification of LDL cholesterol on the association between the KIF6 719Arg allele and the risk of CVD must be exactly equal to the effect modification of KIF6 on the association between LDL cholesterol and the risk of CVD. The equivalence of these estimates of effect modification can also be seen graphically in Figure 4. Additional studies, however, directly comparing the effect of LDL cholesterol on the risk of CVD separately among KIF6 carriers and non-carriers should be performed to validate our findings.

In conclusion, we found that the common KIF6 719Arg allele appears to increase vulnerability to LDL cholesterol, and thereby influences the expected clinical benefit from therapies that lower serum LDL cholesterol levels. More generally, our study demonstrates that a common polymorphism can influence vulnerability to a common modifiable risk factor for CVD even if it is not independently associated with the risk of CVD. Our study provides a roadmap for further research that could potentially lead to the discovery of polymorphisms that cause large differences in vulnerability to common modifiable risk factors for CVD and thus lead to correspondingly large differences in the clinical benefit derived from therapies used to treat these risk factors. Incorporating such polymorphism into the care of individual patients may help to guide the selection of optimal medical therapies for each individual person based on their genetic background and therefore introduce a practical clinical strategy to personalize the prevention of CVD.

## Supporting Information

Table S1 A list of studies included in the meta-analysis of the KIF6 719Arg allele and the risk of cardiovascular disease. The included studies are Atherosclerotic Disease, Vascular Function, and Genetic Epidemiology study (ADVANCE); Acute Myocardial Infarction Gene Study, Dortmund Health Study (AMI Gene Study); CATHGENE Research Project (CATHGENE); deCODE CAD Study (deCODE); National FINRISK studies (FINRISK); German Myocardial Infarction Family Study I (GerMIFS I); German Myocardial Infarction Family Study II (GerMIFS II); Heart Attack in Puget Sound study (HARPS); International Heart Study (INTERHEART); Malmo Diet and Cancer Study (MDS); Washington Hospital Center catheterization study (MEDSTAR); Massachusetts General Hospital study of premature CAD (MGH PCAD); Mid-America Heart Institute (MAHI); Penn-CATH study; Registre Gironi de Cor (REGIGOR); Verona Heart Study (VHS); Wellcome Trust Case Control Consortium CAD Study (WTCCC); Ottawa Heart Study; Cholesterol and Recurrent Events (CARE); West of Scotland Coronary Prevention Study (WOSCOPS); Pravastatin or Atorvastatin Evaluation and Infection Therapy: Thrombolysis in Myocardial Infarction 22 (PROVE-IT TIMI22); Prospective Study of Pravastatin in the Elderly at Risk (PROSPER); Heart Protection Study (HPS); Justification for the Use of Statins in Primary Prevention trial: An Intervention Trial Evaluating Rosuvastatin (JUPITER); Treat to New Targets (TNT); Incremental Decrease in Events through Aggressive Lipid Lowering (IDEAL); Atherosclerosis Risk in Communities study (ARIC); Cardiovascular Health Study (CHS); Women's Health Study (WHS).
(PDF)

Table S2 A description of the baseline characteristics of the randomized trials included in the meta-analysis of the clinical benefit of statin therapy stratified by KIF6 carrier status. The Difference in average LDL (mmol/L) is the difference between either treatment allocation arm. Study acronyms are defined in Table S1.
(PDF)

Text S1 Equivalent measures of effect modification.
(PDF)
