# Supplementary material for: A Common KIF6 Polymorphism Increases Vulnerability to Low-Density Lipoprotein Cholesterol: Two Meta-Analyses and a Meta-Regression Analysis
Source: PLoS One. 2011 Dec 21;6(12):e28834. doi: 10.1371/journal.pone.0028834 (PMC3244415; doi:10.1371/journal.pone.0028834)
Supplement: Table S1 — A list of studies included in the meta-analysis of the KIF6 719Arg allele and the risk of cardiovascular disease. The included studies are Atherosclerotic Disease, Vascular Function, and Genetic Epidemiology study (ADVANCE); Acute Myocardial Infarction Gene Study, Dortmund Health Study (AMI Gene Study); CATHGENE Research Project (CATHGENE); deCODE CAD Study (deCODE); National FINRISK studies (FINRISK); German Myocardial Infarction Family Study I (GerMIFS I); German Myocardial Infarction Family Study II (GerMIFS II); Heart Attack in Puget Sound study (HARPS); International Heart Study (INTERHEART); Malmo Diet and Cancer Study (MDS); Washington Hospital Center catheterization study (MEDSTAR); Massachusetts General Hospital study of premature CAD (MGH PCAD); Mid-America Heart Institute (MAHI); Penn-CATH study; Registre Gironi de Cor (REGIGOR); Verona Heart Study (VHS); Wellcome Trust Case Control Consortium CAD Study (WTCCC); Ottawa Heart Study; Cholesterol and Recurrent Events (CARE); West of Scotland Coronary Prevention Study (WOSCOPS); Pravastatin or Atorvastatin Evaluation and Infection Therapy: Thrombolysis in Myocardial Infarction 22 (PROVE-IT TIMI22); Prospective Study of Pravastatin in the Elderly at Risk (PROSPER); Heart Protection Study (HPS); Justification for the Use of Statins in Primary Prevention trial: An Intervention Trial Evaluating Rosuvastatin (JUPITER); Treat to New Targets (TNT); Incremental Decrease in Events through Aggressive Lipid Lowering (IDEAL); Atherosclerosis Risk in Communities study (ARIC); Cardiovascular Health Study (CHS); Women's Health Study (WHS). (PDF) [file pone.0028834.s001.pdf]

Table S1: Studies included in the meta-analysis of the KIF6 719Arg allele and the risk of cardiovascular disease

Study	Definition of "case" status	Number of Participants	Number of CVD Events (cases)
**ADVANCE**	Acute MI	586	275
**AMI Gene Study**	Acute MI	1,914	793
**CATHGEN**	MI	2,028	1,298
**deCODE**	MI or coronary revascularization	29,265	4,313
**FINRISK**	MI	339	167
**GerMIFS I**	MI	2,365	722
**GerMIFS II**	MI	2,403	1,126
**HARPS**	MI	1,064	505
**INTERHEART**	MI	1,648	789
**MDS**	MI	185	86
**MedStar**	coronary artery ≥ 50% stenosis	1,322	875
**MGH PCAD**	MI	464	204
**MAHI**	History of acute coronary syndrome	1,444	807
**Penn-CATH**	coronary artery ≥ 50% stenosis	1,401	933
**REGICOR**	MI	629	312
**VHS**	coronary artery ≥ 50% stenosis	1,489	1,106
**WTCCC**	MI or CAD	4,855	1,922
**Ottawa Heart Study**	coronary artery ≥ 50% stenosis	2,995	1,540
**CARE (placebo arm)**	MI	1,333	162
**CARE (statin arm)**	MI	1,364	128
**WOSCOPS (placebo arm)**	CHD death or MI	795	276
**WOSCOPS (statin arm)**	CHD death or MI	732	189
**PROVE-IT TIMI 22 (less intense statin arm)**	Death, MI, arterial revascularization, or hospitalization for ACS	907	233
**PROVE-IT TIMI 22 (more intense statin arm)**	Death, MI, arterial revascularization, or hospitalization for ACS	908	177
**PROSPER (placebo arm)**	CHD death, MI, coronary revascularization	2,882	379
**PROSPER (statin arm)**	CHD death, MI, coronary revascularization	2,870	315
**HPS (placebo arm)**	CHD death, MI, stroke, arterial revascularization	9,181	2,335
**HPS (statin arm)**	CHD death, MI, stroke, arterial revascularization	9,167	1,850
**JUPITER (placebo arm)**	CHD death, MI, stroke, arterial revascularization, hospitalization for unstable angina	2,311	207
**JUPITER (statin arm)**	CHD death, MI, stroke, arterial revascularization, hospitalization for unstable angina	2,288	174
**TNT (less intense statin arm)**	CHD death, MI, stroke, resuscitated cardiac arrest	3,305	349
**TNT (more intense statin arm)**	CHD death, MI, stroke, resuscitated cardiac arrest	3,236	299
**IDEAL (less intense statin arm)**	CHD death, MI, resuscitated cardiac arrest	4,377	146
**IDEAL (more intense statin arm)**	CHD death, MI, resuscitated cardiac arrest	4,404	88
**ARIC**	CHD death, MI, coronary revascularization	9,541	1,000
**CHS**	MI	3,651	395
**WHS**	CHD death, MI, stroke, coronary revascularization	25,283	990
**TOTAL**		144,931	27,465
