# Supplementary material for: A Common KIF6 Polymorphism Increases Vulnerability to Low-Density Lipoprotein Cholesterol: Two Meta-Analyses and a Meta-Regression Analysis
Source: PLoS One. 2011 Dec 21;6(12):e28834. doi: 10.1371/journal.pone.0028834 (PMC3244415; doi:10.1371/journal.pone.0028834)
Supplement: Table S2 — A description of the baseline characteristics of the randomized trials included in the meta-analysis of the clinical benefit of statin therapy stratified by KIF6 carrier status. The Difference in average LDL (mmol/L) is the difference between either treatment allocation arm. Study acronyms are defined in Table S1. (PDF) [file pone.0028834.s002.pdf]

Table S2: Baseline characteristics of studies included in the meta-analysis of the clinical benefit of statin therapy stratified by KIF6 carrier status

Study	Genetic Sub-Study Sample Size	Number of CVD Events	Treatment Comparison	Primary Outcome	Median Follow-up (y)	[1]Difference in average LDL (mmol/L)
**CARE**	2,697	290	Statin vs. placebo	CHD death or MI	**5.0**	**-0.98**
**WOSCOPS**	1,227	465	Statin vs. placebo	CHD death or MI	**4.8**	**-1.16**
**PROVE-IT TIMI22**	1,815	410	More intense statin vs. less intense statin	Death, MI, arterial revascularization, or hospitalization for ACS	**2.1**	**-0.85**
**PROSPER**	5,752	694	Statin vs. placebo	CHD death, MI, coronary revascularization	**3.3**	**-1.04**
**HPS**	18,348	4,185	Statin vs. placebo	CHD death, MI, stroke, arterial revascularization	**5.4**	**-1.00**
**JUPITER**	4,599	381	Statin vs. placebo	CHD death, MI, stroke, arterial revascularization, hospitalization for unstable angina	**2.0**	**-1.09**
**TNT**	6,541	648	More intense statin vs. less intense statin	CHD death, MI, stroke, resuscitated cardiac arrest	**5.0**	**-0.62**
**IDEAL**	8,781	234	More intense statin vs. less intense statin	CHD death, MI, resuscitated cardiac arrest	**4.8**	**-0.59**
**TOTAL**	**50,060**	**7,307**				

[1]Difference in average LDL (mmol/L) between either treatment allocation arm. Study acronyms are defined in Table S1.

Abbreviations: CHD: Coronary heart disease; MI: myocardial infarction; ACS: acute coronary syndrome.
