# Supplementary material for: A Common KIF6 Polymorphism Increases Vulnerability to Low-Density Lipoprotein Cholesterol: Two Meta-Analyses and a Meta-Regression Analysis
Source: PLoS One. 2011 Dec 21;6(12):e28834. doi: 10.1371/journal.pone.0028834 (PMC3244415; doi:10.1371/journal.pone.0028834)
Supplement: Text S1 — Equivalent measures of effect modification. (PDF) [file pone.0028834.s003.pdf]

SUPPORTING INFORMATION

A Common KIF6 Polymorphism Increases Vulnerability to Low-Density Lipoprotein Cholesterol: Two Meta-Analyses and a Meta-Regression Analysis

Brian A. Ference, M.D., M.Phil., M.Sc., Wonsuk Yoo, Ph.D., John M. Flack, M.D., M.P.H., Michael Clarke, D.Phil.

Text S1: Equivalent Measures of Effect Modification

For simplicity and clarity of presentation, the following refers to the effect modification between two dichotomous exposure variables, A and B, and the risk of a dichotomous outcome (DIS). Extension to continuous variables is straightforward.

A) Measuring Effect Modification in a Generalized Linear Model

For the following generalized linear model (logistic regression model) containing an interaction term:

$$Ln(Odds_{DIS}) = constant + ß_1(A) + ß_2(B) + ß_3(A*B)$$

The interpretation of each term in the model is straightforward:

- $e^{ß1}$ = OR $(A\text{-}DIS)_{B=0}$; is the Odds Ratio (OR) measuring the association between exposure A and the risk of disease (DIS), among persons not exposed to B (B=0)
- $e^{ß2}$ = OR $(B\text{-}DIS)_{A=0}$; is the OR measuring the association between exposure B and the risk of disease, among persons not exposed to A (A=0)
- $e^{ß3}$ = OR (A-B interaction); is the OR for the interaction, or effect modification, between exposures A and B

To obtain the OR for the association between exposure A and the risk of disease among persons exposed to B ($OR_{(A\text{-}DIS)\ B=1}$), the OR for the association between exposure A and the risk of disease among persons not exposed to B ($OR_{(A\text{-}DIS)\ B=0}$) is multiplied by the interaction OR:

$$OR_{(A\text{-}DIS)\ B=1} = OR_{(A\text{-}DIS)\ B=0} \times OR_{(A\text{-}B\ interaction)}$$

With re-arrangement:

$$[1] \qquad OR_{(A\text{-}B\ interaction)} = \frac{OR_{(A\text{-}DIS)\ B=1}}{OR_{(A\text{-}DIS)\ B=0}}$$

The interaction OR is thus the ratio of the OR's for the association between exposure A and the risk of disease among persons exposed to B and not exposed to B, respectively.

Similarly, to obtain the OR for the association between exposure B and the risk of disease among persons exposed to A ($OR_{(B\text{-}DIS)\ A=1}$), the OR for the association between exposure B and the risk of disease among persons not exposed to A ($OR_{(B\text{-}DIS)\ A=0}$) is multiplied by the interaction OR:

$$OR_{(B\text{-}DIS)\ A=1} = OR_{(B\text{-}DIS)\ A=0} \times OR_{(A\text{-}B\ interaction)}$$

With re-arrangement:

$$[2] \qquad OR_{(A\text{-}B\ interaction)} = \frac{OR_{(A\text{-}DIS)\ B=1}}{OR_{(A\text{-}DIS)\ B=0}}$$

The interaction OR is thus <u>also</u> the ratio of the OR's for the association between exposure B and the risk of disease among persons exposed to A and not exposed to A, respectively.

Therefore, in a generalized linear model, a single interaction term defines the effect modification between any two exposure variables. As a result, the effect modification of exposure B on the association between exposure A and the risk of disease is exactly

equal to the effect modification of exposure A on the association between exposure B and the risk of disease, because these two estimates of effect modification are defined by the same interaction term as shown below:

$$[3] \quad \frac{OR_{(A\text{-}DIS)\ B=1}}{OR_{(A\text{-}DIS)\ B=0}} = OR_{(A\text{-}B\ interaction)} = \frac{OR_{(B\text{-}DIS)\ A=1}}{OR_{(B\text{-}DIS)\ A=0}}$$

.

B) Measuring Effect Modification in a Meta-Regression Equation

The regression term in a meta-regression equation is also a measure of effect modification, and it is equivalent to an interaction term in a generalized linear model.

The following meta-regression equation estimates the effect modification of exposure B on the association between exposure A and the risk of disease (using study level values for the included variables):

$$\ln(OR_{(A\text{-}DIS)}) = \text{constant} + ß_1(B)$$

When B=0, that is in studies conducted among persons not exposed to B:

$$Ln(OR_{(A\text{-}DIS)\ B=0}) = \text{constant} + ß_1 {*}(0), \text{ or}$$

$$Ln(OR_{(A\text{-}DIS)\ B=0}) = \text{constant}$$

Taking the inverse natural log of both sides

$$OR_{(A\text{-}DIS)\ B=0} = e^{\text{constant}}$$

Therefore, the inverse natural logarithm of the constant term in this meta-regression equation is the OR measuring the association between exposure A and the risk of disease, *among persons not exposed to B (B=0).*

When B=1, that is in studies conducted among persons exposed to B:

$$Ln(OR_{(A\text{-}DIS)\ B=1}) = constant + ß_1*(1), \text{ or}$$

$$Ln(OR_{(A\text{-}DIS)\ B=1}) = constant + ß_1$$

Taking the inverse natural log of both sides (and recalling that the inverse natural log of the constant term is the OR for the association between exposure A and the risk of disease when B=0),

$$OR_{(A\text{-}DIS)\ B=1} = OR_{(A\text{-}DIS)\ B=0} \times OR_{(Effect\ Modification\ of\ B)}$$

Thus the OR for the association between exposure A and the risk of disease among persons exposed to B ($OR_{(A\text{-}DIS)\ B=1}$) is equal to the OR for the association between exposure A and the risk of disease among persons not exposed to B ($OR_{(A\text{-}DIS)\ B=0}$) multiplied by the OR for the meta-regression term estimating the effect modification of exposure B on the association between exposure A and the risk of disease.

With re-arrangement,

$$[4] \qquad OR_{(Effect\ Modification\ of\ B)} = \frac{OR_{(A\text{-}DIS)\ B=1}}{OR_{(A\text{-}DIS)\ B=0}}$$

Therefore, the inverse natural logarithm of the regression term in this meta-regression equation is the ratio of the OR's for the association between exposure A and the risk of disease among persons exposed to B and not exposed to B, respectively. This is exactly the same interpretation as for the interaction term in the logistic model evaluating the interaction between exposures A and B as shown in equation [1].

Combining equations [1] through [4], we see that:

$$[5] \qquad OR_{(Effect\ Modification\ of\ B)} = \frac{OR_{(A\text{-}DIS)\ B=1}}{OR_{(A\text{-}DIS)\ B=0}} = OR_{(A\text{-}B\ interaction)}$$

Thus a regression term in a meta-regression equation and an interaction term in a generalized linear model are merely different methods of estimating the same effect modification between any two exposures, and these two measures of effect modification are conceptually and numerically equivalent.

C) Equivalent Measures Effect Modification

We have shown that a single interaction term in a generalized linear model defines the effect modification between any two exposure variables, and as a result the estimate of the effect modification of exposure B on the association between exposure A and the risk of disease is exactly equal to the estimate of the effect modification of exposure A on the association between exposure B and the risk of disease, as shown in equation [3]. We have also shown that a regression term in a meta-regression equation and an interaction term involving the same two variables in a generalized linear model are equivalent measures of effect modification, as shown in equation [5]. Therefore, like the interaction term in generalized linear model, the regression term in a meta-regression equation must also provide a simultaneous estimate of BOTH the effect modification of exposure B on the association between exposure A and the risk of disease AND an estimate of the effect modification of exposure A on the association between exposure B and the risk of disease, because these two estimates of effect modification are equal as shown below:

$$[6] \qquad OR_{(\text{Effect Modification of B})} = \frac{OR_{(\text{A-DIS})\ \text{B=1}}}{OR_{(\text{A-DIS})\ \text{B=0}}} = OR_{(\text{A-B interaction})} = \frac{OR_{(\text{B-DIS})\ \text{A=1}}}{OR_{(\text{B-DIS})\ \text{A=0}}}$$
